# Sex differences in in-hospital mortality following a first acute myocardial infarction: symptomatology, delayed presentation, and hospital setting

**DOI:** 10.1186/s12872-016-0276-5

**Published:** 2016-05-26

**Authors:** George Mnatzaganian, George Braitberg, Janet E. Hiller, Lisa Kuhn, Rose Chapman

**Affiliations:** School of Allied Health, Faculty of Health Sciences, Australian Catholic University, Fitzroy, Victoria 3065 Australia; Department of Medicine, The University of Melbourne, Parkville, Victoria 3010 Australia; Department of Emergency Medicine, Royal Melbourne Hospital, Parkville, Victoria 3010 Australia; School of Health Sciences, Faculty of Health, Arts and Design, Swinburne University of Technology, Hawthorn, Victoria 3122 Australia; Discipline of Public Health, School of Population Health, The University of Adelaide, Adelaide, South Australia 5000 Australia; School of Nursing and Midwifery, Faculty of Health, Deakin University, Geelong, Victoria 3220 Australia; School of Physiotherapy and Exercise Science, Faculty of Health Sciences, Curtin University, Bentley, Western Australia 6102 Australia

**Keywords:** Attributable risk, Hospital setting, In-hospital mortality, Myocardial infarction, Sex disparity

## Abstract

**Background:**

Women generally wait longer than men prior to seeking treatment for acute myocardial infarction (AMI). They are more likely to present with atypical symptoms, and are less likely to be admitted to coronary or intensive care units (CCU or ICU) compared to similarly-aged males. Women are more likely to die during hospital admission. Sex differences in the associations of delayed arrival, admitting ward, and mortality have not been thoroughly investigated.

**Methods:**

Focusing on presenting symptoms and time of presentation since symptom onset, we evaluated sex differences in in-hospital mortality following a first AMI in 4859 men and women presenting to three emergency departments (ED) from December 2008 to February 2014. Sex-specific risk of mortality associated with admission to either CCU/ICU or medical wards was calculated after adjusting for age, socioeconomic status, triage-assigned urgency of presentation, blood pressure, heart rate, presenting symptoms, timing of presentation since symptom onset, and treatment in the ED. Sex-specific age-adjusted attributable risks were calculated.

**Results:**

Compared to males, females waited longer before seeking treatment, presented more often with atypical symptoms, and were less likely to be admitted to CCU or ICU. Age-adjusted mortality in CCU/ICU or medical wards was higher among females (3.1 and 4.9 % respectively in CCU/ICU and medical wards in females compared to 2.6 and 3.2 % in males). However, after adjusting for variation in presenting symptoms, delayed arrival and other risk factors, risk of death was similar between males and females if they were admitted to CCU or ICU. This was in contrast to those admitted to medical wards. Females admitted to medical wards were 89 % more likely to die than their male counterparts. Arriving in the ED within 60 min of onset of symptoms was not associated with in-hospital mortality. Among males, 2.2 % of in-hospital mortality was attributed to being admitted to medical wards rather than CCU or ICU, while for females this age-adjusted attributable risk was 4.1 %.

**Conclusions:**

Our study stresses the need to reappraise decision making in patient selection for admission to specialised care units, whilst raising awareness of possible sex-related bias in management of patients diagnosed with an AMI.

## Background

Cardiovascular disease (CVD), of which coronary heart disease (CHD) accounts for more than half, is a global health problem that contributes considerably to global mortality and disease burden in men and women [1–5]. In the United States, it accounts for 1 in 2.8 deaths per year [2], while in the United Kingdom, approximately 1 in 6 deaths in men and 1 in 10 in women each year are from CHD [1, 3]. In 2005, from a total of 58 million deaths worldwide, 7.6 million (i.e., 13 %) were due to coronary heart disease [4]. Myocardial infarction (MI) is one of the manifestations of CHD and its incidence in a population is often used as a proxy for estimating the coronary heart disease burden for that country [4]. Each year, it is estimated that around 55,000 Australians suffer an acute myocardial infarction (AMI), on average claiming the lives of 27 individuals each day [5]. Notwithstanding, various reports over the past two decades suggest that mortality from a first AMI is steadily decreasing [6], mainly owing to new technologies, revascularisation, more effective drugs to control for heart related conditions and increased diagnosis of previously indeterminable AMI by high sensitivity blood tests [7]. Nonetheless, females hospitalised with AMI have well-documented higher rates of in-hospital mortality than males [8–10]; which is often attributed to the relatively older age of women at the time of diagnosis compared to men [9]. However, differences in in-hospital mortality following AMI have been mainly observed among younger women compared to their similarly aged male counterparts [10, 11]. Hochman and colleagues have suggested that this may be attributed to the different clinical presentation with which women arrive at hospital and the subsequent greater in-hospital complications [12]. Sex-based disparities have also been reported in the treatment of AMI, and it has been argued that such differences could be associated with sex bias in physicians’ approach to treatment [13]. However, research findings are inconsistent with respect to whether females with AMI are more likely to be undertreated, including the implementation of revascularisation procedures [14–16].

In Australia, as in other countries [17–22], a considerable proportion of patients diagnosed with AMI is treated in medical wards rather than in coronary care units (CCU) or intensive care units (ICU). It has been reported that patients treated in CCU are less likely to die during hospital admission than those treated in medical wards [20, 21], although in some studies, these findings may have been confounded by the older age of patients treated in medical wards [17, 21]. Sex-specific risk of in-hospital mortality attributable to the hospital treatment setting is not known. Our study is unique in that it investigates sex differences in the associations of delayed arrival, admitting ward, and in-hospital mortality after being diagnosed with a first AMI in a large sample of men and women. The study also assesses the sex-specific age-adjusted risk of dying attributed to being admitted to CCU, ICU or medical wards.

## Methods

### Study population

All adult (18 years or older) patients hospitalised after being diagnosed with a first acute myocardial infarction (AMI) in the emergency department of three acute care Victorian teaching hospitals in Australia between 1^st^ December 2008 and 28^th^ February 2014 were eligible to participate in this study. The three participating hospitals serve a large catchment population with a total of approximately 170,000 presentations per annum. All repeat admissions following discharge from the index AMI admission were excluded. Patients were not eligible to participate if they were diagnosed with AMI after experiencing a trauma or any other injury.

### Study variables

Case identification for AMI relied on recorded diagnoses in the emergency department electronic database (SYMPHONY Version 2.29, Ascribe plc, Bolton, UK) using codes from the International Classification of Diseases, 10^th^ Revision, (Australian Modification) (ICD-10-AM). ICD-10-AM codes 121.0-121.9 were used to define AMI. These codes include all myocardial infarctions specified as acute or within a stated duration of 4 weeks or less from onset. These codes do not include old myocardial infarctions or recurrent myocardial infarctions. Data collected for each patient included age, sex, socio-economic status as defined by the Socio Economic Index For Areas (SEIFA) [23], country of birth, language spoken at home, symptoms and main cause of presentation, time from onset of symptoms to arrival to ED, arrival by ambulance, triage urgency score, systolic and diastolic blood pressures, heart rate, time from arrival to the ED to being seen by an attending physician, screening blood tests and electrocardiograms ordered in the ED, length of stay in the ED, hospital ward destination, and in-hospital mortality. All clinical variables relating to the presentation at the ED and the vital signs including blood pressure and pulse rate were measured and recorded by a triage nurse. Vital signs were measured once on arrival to the ED. Atypical symptoms were defined as dyspnoea, diaphoresis, nausea and vomiting, syncope, abdominal pain and musculoskeletal pain.

Socio Economic Index For Areas (SEIFA) is a composite index that ranks geographic areas across Australia according to their relative socio-economic advantage or disadvantage based on Australian Bureau of Statistic 2006 census data, with lower scores indicating more disadvantage.

### Statistical analysis

Patients admitted to hospital were followed until they were discharged, died or were right censored at the end of study (February 28, 2014). The sex-specific analyses were conducted on all patients as a single group and separately on patients 50 years of age or younger and those 51 years of age or older. This cut-point was chosen to differentiate between pre and post-menopausal women in the multicultural Australian population where natural menopause occurs between 45 and 55 years of age [24]. Patients’ characteristics were summarised using descriptive statistics. Pearson chi-square tests were used to compare groups of interest and Student’s *t*-test or ANOVA were used to compare means. Sex differences in age-adjusted in-hospital mortality rates among patients admitted to CCU, ICU or medical wards were analysed using the direct adjustment method – a method that calculates a weighted average of the group’s age-specific mortality rates where the weights represent the age-specific sizes of a standard population [25]. The sex-specific attributable risk (AR) of dying associated with the admitting hospital ward (i.e., CCU or ICU compared to medical wards) was calculated using the following formula:

$$ Attributable\  risk=\frac{\left( Incidence\  in\  total\  population\right)-\left( incidence\  in\  nonexposed\  populaiton\right)}{\mathrm{Incidence}\ \mathrm{in}\ \mathrm{total}\ \mathrm{population}} $$

The weighted-sum approach was utilised to calculate the age-adjusted attributable risk (AR) using the following formula [26]:

$$ AR={\displaystyle \sum_j}wj\ ARj $$

where *AR*_*j*_ and w_*j*_, respectively, denote the AR value specific to different *j* age category levels and the corresponding weight. The weight was calculated as the inverse variance of the AR estimate in each age category over the sum of inverse variances over all age groups [26].

Furthermore, we constructed a multivariable logistic regression to analyse the association between the study variables and in-hospital mortality binary outcome.

### Multiple imputation for missing data

Approximately 2 % of the patients had a missing value on time from onset of symptoms to arrival to the ED, while 39 % had a missing value on vital signs. Multiple imputation technique was used to estimate the missing data as suggested by Rubin [27]. The technique assigned twenty plausible values to the missing data representing the uncertainty about the true value. These multiple imputed datasets were then analysed. The *mi* Stata command was used to conduct the imputations.

### Sensitivity analyses

Uncertainly levels of comorbidity in males and females were accounted for by using sensitivity analysis [28, 29]. Sex-specific uncertainty in the true prevalence of comorbidity, which may differ by sex, was simulated by performing sensitivity analyses with a range of +/− 5 % in the group with AMI while keeping the prevalence of those without AMI constant.

### Assumptions used in the sensitivity analyses

We assumed that the risk-adjusted odds ratio (OR) relating comorbidity (measured as Charlson Comorbidity Index) to in-hospital mortality in patients diagnosed with AMI was 1.17 (95 % CI, 1.11–1.23) as reported in a seven-year longitudinal study that assessed the impact of comorbidities on in-hospital mortality among a similar population of male and female patients diagnosed with AMI [30]. The risk-adjusted OR was derived from a multivariable regression model that accounted for age, sex, Charlson Comorbidity Index, and coronary revascularisation. Comorbidity prevalence in adult hospitalised patients with and without an AMI was set as 35 % [based on a multi-centre ten-year study] [31] and 32 % [based on data from six countries] [32].

All analyses were performed using Stata statistical program (version 13, StataCorp, College Station, TX, USA). Statistical significance was set at a *P* value of < 0.05 (two-sided).

## Results

During the study’s 63-month period, 4859 patients were diagnosed with their first AMI in the three emergency departments and were admitted to hospital, with 3293 (67.8 %) being male and 1566 (32.2 %) being female. Compared to males, females waited longer until they sought medical advice, were more likely to arrive to the ED by ambulance, presented with more atypical symptoms and were more likely to receive less acute triage urgency scores by the triage nurse (Table 1). Delayed arrival to the ED was observed for all female patients who presented with any symptom (i.e., chest pain, atypical symptoms) in any age category compared to their male counterparts (Table 2). Irrespective of age, females also waited significantly longer in the ED until they were examined by a physician [mean 63 min (SD 78) in females compared to mean 43 min (SD 64) in males, *P* < 0.001] and were less likely to be admitted to CCU or ICU compared to males (61 % in females compared to 72 % in males, *P* < 0.001) (Table 1). However, no sex-differences were observed in admission to CCU or ICU in those presenting with atypical symptoms (Table 3). Compared to those admitted to medical wards, patients admitted to CCU or ICU were also significantly younger and were assigned a higher urgency level by the triage nurse. Of those admitted to CCU or ICU, 2.5 % died during their index hospital admission compared to 5.2 % of those treated in medical wards (*P* < 0.001). Crude death rates in either CCU or ICU, or medical wards were significantly higher among females (3.3 % in females versus 2.1 % in males in CCU or ICU, *P* = 0.03; and 7.8 % in females versus 3.5 % in males in medical wards, *P* < 0.001). Adjusting for age did not eliminate the disparities in death rates as shown in Table 4.

**Table 1:** Characteristics of patients diagnosed with their first acute myocardial infarction in the emergency department by sex and age category

Characteristics	Age 50 years or younger		Age 51 years or older	
	Male *N* = 647	Female *N* = 175	Male *N* = 2646	Female *N* = 1391
Age, mean (SD)	43.4 (6.1)	43.6 (5.5)	67.9 (11.0)	73.8 (11.4)**
Socioeconomic status^a^, %				
Low	43.0	42.3	38.4	37.8
Middle	28.7	26.3	28.5	26.7
High	28.3	31.4	33.1	35.5
Born in Australia, %	51.3	66.3**	43.6	48.2*
Arrived by ambulance, %	54.7	52.6	69.9	76.1**
Arrived within 60 min of onset of symptoms, %	21.2	10.3**	13.3	9.9*
Triage urgency^b^, %				
Resuscitation /Emergency	77.1	65.1	67.0	53.0
All else	22.9	34.9**	33.0	47.0**
Presenting symptom, %				
Chest pain	86.2	84.0	80.1	71.2
Arrhythmia	6.0	5.1	7.4	7.6
Other (e.g., musculoskeletal, SOB)	5.6	9.1	10.7	18.8
Referred due to abnormal finding	2.2	1.7	1.8	2.4**
Pulse pressure, mean (SD)	67.5 (20.8)	63.1 (25.6)*	76.3 (25.2)	80.1 (28.0)**
Heart rate, mean (SD)	81.1 (14.4)	85.5 (17.4)**	81.9 (14.7)	85.0 (17.1)**
Time from arrival till examined by a physician in minutes, mean (SD)	30.9 (52.9)	49.1 (61.9)**	46.1 (65.9)	64.5 (79.4)**
Discharged from ED to: %				
CCU or ICU or operating theatre	78.8	69.1	70.7	60.3
Medical ward	21.2	30.9**	29.3	39.7**

*Abbreviations*: *CCU* coronary care unit, *ED* emergency department, *ICU* intensive care unit, *SD* standard deviation, *SOB* shortness of breath

**P value <0.001; * 0.001 < *P* value < 0.05

^a^The socioeconomic status was based on the Socio-Economic Index For Areas disadvantage score (SEIFA)

^b^The triage score is a ranking from one to five (one being the most urgent and five being non-urgent), given by a Triage nurse, used to prioritise or classify patients on the basis of illness or injury severity and need for medical and nursing care

**Table 2:** Percent arrived to emergency department within 60 min of onset of symptoms by presenting symptom, sex, and age category

	Age 50 years or younger		Age 51 or older	
	Male	Female	Male	Female
Chest pain	20.1	10.2	13.1	9.8
Arrhythmia	46.2	11.1	24.5	18.9
Atypical symptoms (e.g., musculoskeletal pain, shortness of breath)	16.7	12.5	8.2	7.3
Referred to emergency department due to abnormal findings	7.1	0.0	6.3	5.9
All	21.2	10.3	13.3	9.9

**Table 3:** Percent admitted to coronary care unit or intensive care unit by presenting symptom, sex, and age category

	Age 50 years or younger		Age 51 or older	
	Male	Female	Male	Female
Chest pain	79.0	70.1*	74.6	67.0**
Arrhythmia	87.2	66.7	68.9	43.4**
Atypical symptoms (e.g., musculoskeletal pain, shortness of breath)	63.9	56.3	44.7	43.3
Referred to emergency department due to abnormal findings	85.7	100.0^a^	60.4	50.0
All	78.7	69.1*	70.7	60.3**

^a^Less than 5 patients

**P value <0.001; * 0.001 < *P* value < 0.05

**Table 4:** Age adjusted rates of in-hospital mortality among patients admitted to CCU or ICU versus medical hospital wards by sex: direct adjustment method^a^

		Male				Female			
		CCU or ICU		Medical ward		CCU or ICU		Medical ward	
Age groups	Standard Population	Death rate %	Expected # death	Death rate %	Expected # death	Death rate %	Expected # death	Death rate %	Expected # death
All ages	4859								
18–54	1223	0.53	6.5	0.97	11.9	1.10	13.5	2.70	33.0
55–65	1245	1.78	22.2	1.84	22.9	1.72	21.4	1.52	18.9
66–78	1289	2.77	35.7	3.65	47.0	2.95	38.0	3.97	51.2
79 +	1102	5.80	63.9	6.67	73.5	7.08	78.0	12.03	132.6
Total number of deaths expected			128		155		151		236
Crude rates		2.1 %		3.5 %		3.3 %		7.8 %	
Age adjusted rates		128/4859 = 2.6 %		155/4859 = 3.2 %		151/4859 = 3.1 %		236/4859 = 4.9 %	

*Abbreviations*: *CCU* coronary care unit, *ICU* intensive care unit

^a^Direct adjustment method is one that calculates a weighted average of the group’s age-specific mortality rates where the weights represent the age-specific sizes of a standard population

A multivariable logistic regression that also adjusted for age, sex, socioeconomic status, country of birth, language spoken at home, symptoms and main cause of presentation, time from onset of symptoms to arrival to ED, arrival in ambulance, urgency level, heart rate, time from arrival in the ED until examination by attending physician, length of stay in the ED and hospital providing the service, showed that those admitted to CCU or ICU were significantly less likely to die than those admitted to medical wards (adjusted-OR = 0.5, 95 % CI 0.3–0.7, *P* < 0.001) (Table 5). This association was observed in both sexes (CCU/ICU adjusted-OR = 0.5, 95 % CI 0.3–0.9, *P* = 0.013 for males; and 0.4, 95 % CI 0.3–0.7, *P* = 0.003 for females). Compared to those experiencing any of the other presenting symptoms, those presenting with chest pain were 70 % less likely to die during their hospital admission (adjusted-OR = 0.3, 95 % CI 0.2–0.4, *P* < 0.001). Arriving in the emergency department within 60 min of onset of symptoms was not associated with in-hospital mortality (adjusted-OR = 0.9, 95 % CI 0.6–1.9, *P* = 0.9), also observed in both sexes.

**Table 5:** Risk of in-hospital mortality following a diagnosis of first acute myocardial infarction: a multivariable logistic regression^a^

Covariate	Odds ratio, 95 % CI	*P* value
Age categories (tertiles)		
18–58 years (reference)	1.00	
59–74 years	2.3 (1.3–4.3)	0.006
75 year or more	5.0 (2.8–9.1)	<0.001
Female sex	1.6 (1.1–2.3)	0.007
Socioeconomic status^b^		
Low tertile (reference)	1.00	
Middle tertile	0.8 (0.5–1.3)	0.4
High tertile	0.8 (0.6–1.2)	0.3
Born in Australia	0.8 (0.6–1.1)	0.2
Chest pain as main presenting symptom	0.3 (0.2–0.4)	<0.001
Arrival in the ED within 60 min of onset of symptoms	0.9 (0.6–1.6)	0.9
Arrival in ambulance	3.9 (2.0–7.8)	<0.001
Triage classification of urgency		
Non-urgent presentations (reference)	1.00	
Emergency presentations/resuscitation needed	3.0 (1.8–4.9)	<0.001
Admitted to CCU or ICU	0.5 (0.3–0.7)	<0.001
Time from arrival in the ED to examination by physician (continuous variable)	1.0 (0.9–1.0)	0.9

*Abbreviation*: *CCU* coronary care unit, *CI* confidence interval, *ED* emergency department, *ICU* intensive care unit

^a^Also adjusted for hour of presentation, hospital type, length of stay in the ED, and language spoken at home

^b^The socioeconomic status was based on the Socio-Economic Index For Areas disadvantage score (SEIFA)

Running separate multivariable models by hospital treating ward showed that females admitted to medical wards were 89 % more likely to die than their male counterparts (adjusted-OR = 1.89, 95 % CI 1.1–3.2, *P* = 0.017), but no statistically significant sex differences were observed in risk of dying in CCU or ICU (adjusted-OR = 1.5, 95 % CI 0.9–2.4, *P* = 0.1).

After adjusting for age, among males, 2.2 % of in-hospital mortality was attributed to being admitted to medical wards compared with admission to CCU or ICU, whereas in females, this age-adjusted attributable risk was 4.1 %. Accounting for various levels of comorbidity in males and females using sensitivity analyses did not eliminate gender differences in in-hospital mortality (Appendix).

## Discussion

In this 5.3-year study, we have shown that a considerable amount of sex disparity in in-hospital mortality after being admitted with a first AMI is attributed to the hospital admitting ward. In medical wards, women in our study had worse short-term outcomes with higher in-hospital mortality rates than men, which could not be explained by their age, presenting symptoms, delayed arrival, or treating hospital. Sensitivity analyses that accounted for uncertainty in comorbidity also showed similar results. No such sex differences were observed among those admitted in coronary or intensive care units. Based upon our results, admitting patients who are diagnosed with AMI in the ED, to CCU or ICU instead of medical wards could prevent 2 to 4 % of in-hospital mortality.

Although men are more likely than women to be diagnosed with AMI during their lifespan, the short-term outcomes such as mortality are worse for some groups of women, particularly younger women relative to their similarly-aged male counterparts [10]. Some authors argue that the patients’ risk factor profiles may account for much of the sex disparity in in-hospital mortality [9, 10], while others debate that under-treatment of women may be an independent contributor to the sex disparity in mortality [14]. Our study shows that the hospital setting to which the patient is admitted is an important contributor to the sex disparity in in-hospital deaths amongst those diagnosed with AMI. Although admission to CCU or ICU is not without risks [33, 34], we show that such an admission is beneficial to both young and older patients diagnosed with their first AMI, regardless of sex. Patients diagnosed with AMI may often suffer from life-threatening complications that require highly specialised care. Consequently, the standard of care for such patients is to treat them in either coronary or intensive care units, which have been shown to be cost-effective [35]. However, the cost-effectiveness of treatment in the CCU is debatable and less favourable among younger patients because of their relative lower underlying risk of life-threatening complications [36].

Similar to other researchers [37], we found that women with AMI were less often admitted to coronary or intensive care units than their male counterparts. This was mainly observed in those presenting with chest pain or arrhythmia. In the former, admission to these specialised units was less in both younger and older women. The reason for this is unclear, especially because all our patients were diagnosed with AMI before the decision was made about the admitting hospital setting. Furthermore, since the demand for intensive and coronary care unit beds far exceeds their availability [18], patients presenting with symptoms other than chest pain may be more likely to be admitted to medical wards, as was shown in our study. Our analysis indicates that due consideration of the impact of hospital setting must be given when deciding whether to admit a patient with an AMI to a coronary or intensive care unit bed versus a medical ward bed. Since increasing evidence indicates sex disparity in short-term outcomes following AMI, there is a need to re-assess the cost-effectiveness of treatment in specialised CCU and ICU wards, armed with the knowledge that sex and age are contributing factors to in-hospital mortality.

Timely treatment of men and women with acute myocardial infarction depends on symptom recognition and prompt presentation to the emergency department. Although the benefits of early treatment of AMI are clearly evident, only a small proportion of such patients receives treatment within evidence-based timeframes from symptom onset [38]. Our study did not show any significant association between a timely arrival to the hospital and in-hospital mortality. This may be explained by the fact that those who delayed seeking medical advice may have not arrived at the ED, as it has been shown that approximately half of those who have an AMI die before reaching a hospital within an hour of symptom onset [39].

Strengths of our large sample study include our ability to account for presenting symptoms and time to arrival to the ED since onset of symptoms – factors which are often not considered when assessing short-term outcomes following an AMI. The study has some limitations. We have not separately examined whether the effect of the hospital setting is related to the nature of the ward structure (including such factors as nurse-patient ratios, the skill set of the attendant nursing staff or the monitoring capability of the ward) or whether it is related to medical expertise or variation in clinical practices within each of these three hospitals. The physiologic and clinical data were collected once on arrival to the ED and these were not validated; however these were measured and recorded by a triage nurse. Case identification was retrospective being entirely based on the final diagnosis reached at discharge from the ED. Available data did not permit us to validate the ED diagnosis of AMI, as we did not have access to patients’ hospital discharge charts. We also did not have information on patients’ comorbidities. Nevertheless, age, which is often considered the simplest co-morbidity score [40–42], was accounted for over the study period. Similarly, using sensitivity analyses we accounted for various prevalence estimates of comorbidities among males and females, showing similar results in sex differences. Finally, we did not have information on the location (e.g., inferior versus anterior wall MI) or the nature of the myocardial infarction whether it was with ST segment elevation (STEMI) or without (NSTEMI)); however, there is no evidence to indicate that misclassifications of diagnoses such as AMI are sex specific.

## Conclusion

Our study demonstrates that sex differences exist in frequency of admission to coronary or intensive care units among patients diagnosed with an acute myocardial infarction. However, among those admitted to these specialised units, no sex differences were observed in in-hospital mortality after accounting for age, presenting symptoms, delayed arrival and other risk factors. The disparity in this short-term outcome was prominent in the medical wards and was not explained by the patient age or other risk factors. This study calls for appropriate knowledge transfer while addressing decision making regarding admission to coronary or intensive care units while considering possible sex-related biases.

## Abbreviations

AMI, acute myocardial infarction; AR, attributable risk; CCU, coronary care unit; CHD, coronary heart disease; CI, confidence interval; CVD, cardiovascular disease; ED, emergency department; ICD-10-AM, International Classification of Diseases (Australian Modification); ICU, intensive care unit; NSTEMI, Non-ST-segment elevation myocardial infarction; OR, odds ratio; SD, standard deviation; SEIFA, Socio Economic Index For Areas; STEMI, ST-segment elevation myocardial infarction.

## Appendix

**Table 6:** Observed and expected gender-specific odds ratios of in-hospital mortality following acute myocardial infarction: sensitivity analysis accounting for sex-specific uncertainty in the prevalence of comorbidity

Level of uncertainty^a^	Comorbidity prevalence %, females : males	Odds ratio of females dying compared to males	Expected percent bias
		Observed unadjusted OR	–
–	–	2.1 (1.5–2.8)	
		Expected comorbidity adjusted OR: sensitivity analysis^b^	
5 %	40 : 35	2.06	1 %
10 %	45 : 35	2.05	2 %
25 %	60 : 35	2.00	4 %
45 %	80 : 35	1.94	7 %
65 %	100 : 35	1.88	10 %

^a^Uncertainty in the prevalence of comorbidity in females. The assumed prevalence of both females and males was set at 35 % (McManus et al. [31]). The prevalence of comorbidity in females was increased in accordance to the level of uncertainty

^b^The sensitivity analysis evaluated the odds ratio while accounting for unmeasured confounding by comorbidity
